# Feasibility of Artificial Intelligence Models for Longitudinal CT Analysis of Epicardial Adipose Tissue After Immunotherapy

**DOI:** 10.3390/diagnostics16060852

**Published:** 2026-03-13

**Authors:** Eliodoro Faiella, Stefania Lamja, Rebecca Casati, Michele Tondo, Raffaele Ragone, Adriano Redi, Elva Vergantino, Bruno Beomonte Zobel, Francesco Grasso, Domiziana Santucci

**Affiliations:** 1 Fondazione Policlinico Campus Biomedico di Roma, 00128 Roma, Italy; rebecca.casati@policlinicocampus.it (R.C.); raffaele.ragone@policlinicocampus.it (R.R.); adriano.redi@policlinicocampus.it (A.R.); b.zobel@policlinicocampus.it (B.B.Z.); r.grasso@policlinicocampus.it (F.G.); d.santucci@policlinicocampus.it (D.S.); 2 Università Campus Biomedico di Roma, 00128 Roma, Italy; michele.tondo@unicampus.it (M.T.); elva.vergantino@unicampus.it (E.V.); 3 Research Unit of Interventional Radiology, Department of Radiology, 00128 Roma, Italy

**Keywords:** epicardial adipose tissue, artificial intelligence, cardiovascular imaging

## Abstract

**Background**: Epicardial adipose tissue (EAT) is an imaging-derived biomarker increasingly associated with cardiovascular inflammation and metabolic risk. Computed tomography (CT) allows for accurate volumetric quantification of EAT, but the clinical interpretation of longitudinal changes remains challenging. Artificial Intelligence (AI) may provide additional value by identifying patterns and predictors of EAT variation. **Purpose**: To evaluate longitudinal changes in CT-derived EAT volume and to assess the feasibility and performance of AI-based models in discriminating patients with EAT increase after immunotherapy. **Methods**: In this retrospective single-center study, EAT was volumetrically segmented on baseline and follow-up CT scans. EAT change (ΔEAT) was calculated, and patients were dichotomized according to EAT increase (ΔEAT > 0). Three supervised AI models—Support Vector Machine (SVM), Artificial Neural Network (ANN), and Extreme Gradient Boosting (XGBoost)—were trained using imaging-derived and clinical variables. Given the limited sample size and class imbalance, stratified two-fold cross-validation was adopted. Model performance was assessed using AUC, accuracy, and F1-score, and model interpretability was explored using permutation importance. **Results**: EAT volume showed a statistically significant increase at follow-up. In the AI analysis, SVM and ANN demonstrated good discriminative performance, with ANN achieving the highest AUC (~0.90). XGBoost failed to show meaningful predictive capability. Baseline EAT volume and follow-up duration emerged as the most relevant features. **Conclusions**: AI-based models, particularly SVM and ANN, are feasible tools for the analysis of CT-derived EAT changes, even in small cohorts. These results support the integration of AI-assisted EAT assessment into imaging-based cardio-oncology research.

## 1. Introduction

Epicardial adipose tissue (EAT) is a visceral fat depot located between the myocardium and the visceral pericardium, sharing the same microcirculation with the underlying myocardium and lacking a fascial boundary. This close anatomical and functional relationship allows for a direct paracrine and vasocrine interaction between EAT and the heart. Unlike subcutaneous fat, EAT exhibits high metabolic activity and secretes a wide range of bioactive molecules, including pro-inflammatory cytokines, adipokines, and pro-atherogenic mediators, which may influence myocardial function, coronary atherosclerosis, and overall cardiovascular risk [1,2,3]. Increasing evidence suggests that EAT acts not merely as a passive fat reservoir but as an active organ involved in cardiovascular pathophysiology [4,5].

From an imaging perspective, EAT has gained increasing attention as a quantitative and reproducible biomarker. Computed tomography (CT) enables accurate, non-invasive volumetric assessment of EAT with high spatial resolution and standardized attenuation thresholds. CT-based EAT quantification is widely available and can be derived not only from dedicated cardiac CT but also from routinely acquired oncologic or chest CT scans, enhancing its potential applicability in real-world clinical settings [6]. While cross-sectional EAT measurements have been extensively investigated and associated with cardiovascular outcomes, metabolic disorders, and systemic inflammation, the clinical interpretation of longitudinal changes in EAT volume remains poorly explored [7]. In particular, the dynamic behavior of EAT over time and its potential modulation by systemic therapies represent an emerging and insufficiently characterized area of research.

Importantly, epicardial adipose tissue has been shown to provide incremental risk information beyond traditional cardiovascular risk factors, reflecting localized inflammatory and metabolic activity that may not be fully captured by systemic biomarkers [8,9].

Immunotherapy is associated with profound immune activation and systemic inflammatory responses, often accompanied by metabolic alterations. These therapies may influence adipose tissue biology, potentially affecting both the quantity and functional properties of EAT [10]. In this context, longitudinal changes in EAT may reflect complex interactions between immune activation, inflammation, metabolism, and cardiovascular homeostasis. However, disentangling these interactions using conventional statistical methods may be challenging, as traditional approaches typically focus on linear associations and average effects. Increased EAT volume has been associated with ischemic heart disease, atrial arrhythmias—particularly atrial fibrillation—and left ventricular diastolic dysfunction, supporting its role as a clinically relevant cardiometabolic biomarker [11].

Artificial intelligence (AI), and machine learning techniques in particular, offer powerful tools to model complex, non-linear, and multivariate relationships among imaging-derived and clinical variables [12,13,14] Importantly, certain AI models are well suited to small and heterogeneous datasets, which are common in early-stage imaging studies and real-world clinical research [15]. By leveraging these methods, AI may provide complementary insights beyond conventional analyses and support the identification of subtle longitudinal patterns in imaging biomarkers [16].

Despite growing interest in EAT quantification, standardized approaches for interpreting longitudinal changes are still lacking, and most available evidence is derived from cross-sectional analyses. This gap limits the translation of EAT measurements into longitudinal risk assessment strategies and highlights the need for methodological frameworks capable of capturing temporal dynamics in imaging biomarkers.

Unlike previous AI-based studies focusing primarily on cross-sectional EAT quantification, the present work specifically investigates longitudinal changes and their modeling in a real-world immunotherapy cohort, thereby addressing a currently underexplored dynamic dimension of EAT assessment.

In this study, we investigate the feasibility of applying AI-based approaches to the analysis of longitudinal CT-derived EAT changes in a real-world cohort of limited size, with the aim of exploring the potential diagnostic value of AI-enhanced imaging biomarkers in this clinical setting.

## 2. Materials and Methods

### 2.1. Study Design and CT Imaging

This retrospective study included consecutive patients with available baseline and follow-up computed tomography examinations acquired after the initiation of immunotherapy. Baseline CT was defined as the examination performed closest to treatment initiation, while follow-up CT corresponded to the first available examination after therapy onset suitable for longitudinal comparison. CT scans were performed according to institutional imaging protocols and included contrast-enhanced acquisitions routinely used for oncologic assessment.

All CT examinations were reviewed to ensure adequate coverage of the cardiac region and sufficient image quality for epicardial adipose tissue (EAT) analysis. Only patients with technically adequate scans at both time points and complete EAT segmentation were included in the final analysis. Exclusion criteria comprised severe motion artifacts, incomplete cardiac coverage, or insufficient image quality precluding reliable adipose tissue delineation. The study was conducted in accordance with institutional standards for retrospective imaging research.

CT acquisition parameters were consistent with institutional oncologic imaging protocols and did not substantially vary across patients in terms of slice thickness and reconstruction algorithms. All examinations provided adequate cardiac coverage suitable for epicardial adipose tissue quantification.

### 2.2. Epicardial Adipose Tissue Quantification

EAT was segmented and quantified volumetrically on non-ECG-synchronized CT scans using attenuation-based thresholds consistent with adipose tissue. Adipose tissue was defined using an attenuation threshold range between −190 and −30 Hounsfield Units. Segmentation included all voxels within the typical fat attenuation range and encompassed the entire adipose compartment enclosed by the visceral pericardium along the full cardiac silhouette, from the pulmonary artery bifurcation to the cardiac apex.

Semi-automated segmentation was performed, followed by manual review and correction when necessary to ensure anatomical accuracy, particularly at the pericardial boundaries. EAT volume was recorded in milliliters at baseline (EAT_pre) and at follow-up (EAT_post). Longitudinal change was calculated as ΔEAT = EAT_post − EAT_pre. To account for interindividual variability in follow-up duration, a normalized metric representing EAT change per month (ΔEAT/month) was additionally derived and used for descriptive analysis.

### 2.3. Feature Definition and Outcome

The primary outcome of the artificial intelligence analysis was binary classification of EAT increase, defined as a positive longitudinal change in EAT volume (ΔEAT > 0). Input features were selected a priori based on clinical relevance and included imaging-derived, temporal, and clinical variables. Imaging-derived features comprised baseline EAT volume, while temporal information included the duration of follow-up between CT examinations. Clinical covariates included age, sex, body mass index, and the presence of cardiometabolic comorbidities.

Missing data were handled within the machine learning pipeline using median imputation to minimize bias while preserving the limited sample size.

A total of 42 patients were included in the artificial intelligence modeling analysis. Patients were dichotomized according to longitudinal EAT increase (ΔEAT > 0 vs. ΔEAT ≤ 0), and class distribution was explicitly reported to ensure transparency regarding dataset balance.

To minimize dimensionality relative to sample size and reduce overfitting risk, only baseline EAT volume was included as imaging-derived input. The total number of input variables was kept low relative to the number of observations to maintain an acceptable feature-to-sample-size ratio and preserve model stability.

Input variables were selected based on established clinical associations between epicardial adipose tissue and cardiometabolic risk factors reported in prior literature. Multicollinearity among clinical variables (e.g., BMI, dyslipidemia, diabetes mellitus) was assessed prior to modeling and did not reveal critical redundancy that would compromise model stability.

### 2.4. Artificial Intelligence Analysis

Three supervised machine learning models were evaluated to explore different modeling strategies. A Support Vector Machine (SVM) with radial basis function kernel was selected for its robustness in small datasets and its ability to capture non-linear decision boundaries. An Artificial Neural Network (ANN) was implemented as a compact multilayer perceptron using an LBFGS solver to enhance numerical stability and reduce overfitting in limited sample sizes. Extreme Gradient Boosting (XGBoost) was included as a representative ensemble tree-based method capable of modeling complex feature interactions.

Feature standardization was applied for SVM and ANN models, while class imbalance was addressed using class weighting strategies. Given the limited number of observations and the imbalance between outcome classes, stratified two-fold cross-validation was employed to maximize data usage while preserving class distribution. Model performance was assessed using the area under the receiver operating characteristic curve (AUC), accuracy, and F1-score. Model interpretability was explored through permutation importance analysis applied to the SVM classifier to identify the relative contribution of input features.

Given the limited sample size, stratified two-fold cross-validation was selected to maximize the number of observations available for training while preserving class distribution. Although this approach may increase variability in performance estimates, it reduces the risk of excessive data fragmentation. Model performance metrics are therefore reported as mean ± standard deviation across folds to reflect variability.

Hyperparameter tuning was limited and predefined to reduce overfitting risk. Feature standardization, median imputation, and class weighting were performed within the cross-validation pipeline to prevent data leakage. No information from validation folds was used during model training or preprocessing steps.

Model performance metrics are reported as mean ± standard deviation across folds. Area under the curve (AUC) values are presented with 95% confidence intervals.

## 3. Results

### 3.1. Pre–Post Comparisons (Expanded Description)

Epicardial adipose tissue (EAT) volume demonstrated a consistent and statistically significant increase between baseline and follow-up CT examinations across the study population. Both mean and median ΔEAT values indicated a positive longitudinal trend, suggesting that EAT remodeling occurred in the majority of patients during the observation period. The concordance between parametric and non-parametric statistical tests supports the robustness of this finding and indicates that the observed change was not driven by outliers or non-normal data distribution.

In contrast, changes in visceral fat volume showed greater dispersion. While the mean increase reached statistical significance using parametric testing, the borderline result observed with the Wilcoxon signed-rank test suggests substantial inter-individual variability. This discrepancy highlights potential differences in the temporal dynamics and biological behavior of epicardial versus visceral adipose tissue compartments.

Pre–post comparisons for epicardial and visceral adipose tissue are reported in Table 1.

### 3.2. Regression Analysis: Absolute vs. Normalized EAT Change

To further detail the determinants of epicardial adipose tissue change, results of the linear regression analysis for absolute ΔEAT are summarized in Table 2.

Linear regression analysis revealed that different determinants emerged depending on whether absolute or time-normalized EAT change was considered. In the model evaluating absolute ΔEAT, follow-up duration represented the dominant explanatory variable, indicating that longer exposure time was associated with greater EAT remodeling. Dyslipidemia status also contributed meaningfully, suggesting a potential interaction between lipid metabolism and epicardial fat dynamics.

When ΔEAT was normalized per month, the explanatory power of the model increased substantially, underscoring the importance of accounting for heterogeneous follow-up intervals. Regression results for normalized EAT change per month are summarized in Table 3.

In this normalized framework, baseline EAT volume and arterial hypertension emerged as the strongest positive contributors, while metabolic comorbidities such as diabetes mellitus and dyslipidemia exhibited inverse associations. These findings suggest that baseline cardiometabolic status may modulate the rate of EAT change rather than its absolute magnitude.

The normalized ΔEAT/month analysis was limited to 13 patients due to incomplete follow-up interval data in the remaining cohort. Given the reduced sample size, these results should be interpreted cautiously, and statistical power is limited.

### 3.3. Artificial Intelligence Performance Across Models

AI-based classification analyses further emphasized differences among modeling strategies. The SVM and ANN models demonstrated consistent and clinically meaningful discriminative performance despite the small and imbalanced dataset. Notably, the ANN implemented with an LBFGS solver achieved the highest overall performance metrics, suggesting superior capacity to capture non-linear feature interactions under constrained conditions.

In contrast, the XGBoost model failed to achieve discriminative performance beyond chance level. This finding underscores the importance of aligning algorithm complexity with dataset size and supports the preferential use of simpler, well-regularized models in small imaging cohorts. Performance metrics of the evaluated AI models are reported in Table 4.

AUC values are reported with 95% confidence intervals calculated across cross-validation folds.

A consolidated summary of longitudinal epicardial adipose tissue changes and artificial intelligence model performance is presented in Table 5.

## 4. Discussion

The present study explores the application of artificial intelligence (AI) techniques to the longitudinal assessment of epicardial adipose tissue quantified on computed tomography, with the aim of identifying patients exhibiting EAT increase after immunotherapy. The results demonstrate two main findings: first, EAT behaves as a dynamic imaging biomarker, showing measurable longitudinal variation; second, AI-based models—particularly Support Vector Machines (SVM) and compact Artificial Neural Networks (ANN)—are capable of discriminating EAT increase even in a small and imbalanced cohort.

### 4.1. Epicardial Adipose Tissue as a Dynamic Imaging Biomarker

EAT has traditionally been investigated as a static parameter, often measured at a single time point and correlated with cardiovascular risk factors or clinical outcomes. However, growing evidence supports the concept of EAT as a metabolically active and plastic tissue, capable of responding to systemic inflammatory, metabolic, and pharmacologic stimuli. From this perspective, longitudinal changes in EAT volume may provide complementary information beyond baseline measurements alone, reflecting dynamic cardiovascular and systemic processes [1].

CT-based volumetric quantification offers a robust and reproducible method to capture these changes over time. In our study, the marked inter-individual variability in ΔEAT highlights the heterogeneous response of epicardial fat to immunotherapy-related systemic effects. Such heterogeneity may be masked by conventional population-level analyses and reinforces the need for analytical approaches capable of identifying subject-specific patterns [8]. This characteristic makes longitudinal EAT an ideal target for AI-driven analysis, which is inherently designed to model complex, multivariate, and non-linear relationships.

### 4.2. Added Value of Artificial Intelligence in EAT Analysis

Traditional statistical methods, including paired comparisons and linear regression models, are effective for detecting overall trends and identifying linear associations. However, they may be limited in capturing higher-order interactions among imaging-derived and clinical variables, particularly in small and heterogeneous datasets. AI-based approaches address this limitation by enabling multivariate pattern recognition without prespecified assumptions regarding data distribution or linearity [8].

In this study, AI models were tasked with a clinically intuitive objective: classification of EAT increase versus non-increase [17,18]. Despite the limited sample size, both SVM and ANN achieved good discriminative performance, suggesting that meaningful patterns are embedded within the available features [19]. These findings support the concept that EAT-related information derived from CT imaging can be effectively leveraged by AI even in early-phase, exploratory imaging studies.

### 4.3. Model Selection in Small Imaging Datasets

A key methodological insight emerging from this work concerns the relationship between dataset size and algorithm choice. Ensemble tree-based methods such as XGBoost are known to perform exceptionally well in large and diverse datasets but may be prone to instability and overfitting in small cohorts. In contrast, SVM and ANN—when appropriately constrained—demonstrated superior robustness in the present setting [20,21,22].

While the limited sample size likely contributed to the suboptimal performance of XGBoost, additional factors may have played a role. Tree-based ensemble methods often require greater heterogeneity and larger datasets to fully exploit feature interactions. Furthermore, pronounced class imbalance and limited feature dimensionality may have constrained the representational advantage typically observed with boosting algorithms.

The strong performance of the ANN implemented with an LBFGS solver is particularly noteworthy. This optimization strategy avoids stochastic gradient descent and internal validation splits, both of which can introduce variability in limited datasets. Similarly, class-weighted SVM mitigated the impact of class imbalance, a frequent challenge in longitudinal imaging research. The near-random performance observed with XGBoost further underscores the importance of tailoring algorithm selection to dataset characteristics rather than relying on model complexity alone.

### 4.4. Interpretability and Feature Relevance

Interpretability remains a critical requirement for the clinical translation of AI models in medical imaging. In this study, permutation importance analysis applied to the SVM model identified baseline EAT volume and follow-up duration as the most influential predictors of EAT increase. This finding is biologically plausible, as baseline EAT reflects the underlying metabolic and inflammatory milieu, while follow-up duration captures the temporal window over which EAT remodeling may occur. The prominence of imaging-derived features further reinforces the central role of CT-based EAT quantification in AI-driven analysis [19,23].

### 4.5. Clinical Implications and Future Perspectives

From a clinical imaging perspective, AI-assisted longitudinal EAT analysis may enhance cardiovascular risk stratification and patient monitoring, particularly in populations exposed to systemic therapies such as immunotherapy. Automated or semi-automated pipelines integrating CT-based segmentation with AI prediction could support early identification of patients undergoing unfavorable cardiometabolic remodeling [23].

From a research standpoint, this study provides a methodological framework for future investigations. It demonstrates that meaningful AI analyses can be conducted even in small cohorts, provided that algorithm selection, validation strategy, and interpretability tools are carefully optimized. This is particularly relevant in emerging fields such as cardio-oncology, where large datasets are often not readily available.

In addition, the use of artificial intelligence for longitudinal imaging biomarker analysis may have relevant implications for personalized medicine. Rather than relying on population-level thresholds, AI-based approaches enable patient-specific pattern recognition, potentially allowing for an individualized assessment of cardiometabolic risk trajectories. In the context of immunotherapy, where treatment-related inflammatory responses may vary substantially among patients, such personalized imaging biomarkers could provide complementary information to traditional clinical and laboratory parameters.

Furthermore, the ability to extract meaningful information from routinely acquired CT scans supports the concept of opportunistic imaging. The integration of AI-assisted EAT analysis into standard oncologic imaging workflows could be achieved without additional radiation exposure or dedicated cardiac imaging protocols, thereby enhancing the clinical value of existing imaging data.

Although classification of ΔEAT > 0 may represent a potential imaging signal of cardiometabolic remodeling in patients undergoing immunotherapy, the present findings should be interpreted as exploratory and hypothesis-generating. The current study does not establish direct clinical decision thresholds but rather demonstrates the feasibility of AI-assisted longitudinal modeling in a real-world cohort. Future larger-scale and outcome-driven investigations are required to determine whether such modeling approaches can meaningfully inform risk stratification or therapeutic strategies in cardio-oncology practice.

### 4.6. Limitations

Several limitations should be acknowledged. The retrospective and single-center design may limit generalizability. The small sample size, although representative of real-world imaging cohorts, precluded external validation and restricted the complexity of the models that could be reliably applied. In addition, the lack of hard cardiovascular outcomes prevents direct assessment of the prognostic implications of EAT increase. Future multicenter studies with larger populations and outcome data are warranted to confirm and extend these findings.

Another limitation relates to the use of non-ECG-synchronized CT scans, which may introduce variability in cardiac phase-dependent measurements. However, previous studies have demonstrated acceptable reproducibility of EAT volumetric quantification on non-gated CT, particularly when semi-automated segmentation and consistent anatomical landmarks are applied. This supports the reliability of the approach adopted in the present study and its applicability in routine clinical practice.

Importantly, no independent test set or external validation cohort was available. Therefore, model performance should be interpreted as exploratory and hypothesis-generating rather than definitive. External validation in larger multicenter cohorts is necessary before clinical translation.

## 5. Conclusions

AI-driven analysis of CT-derived epicardial adipose tissue is feasible and informative, even in small real-world cohorts. In this study, Support Vector Machines and compact Artificial Neural Networks demonstrated robust performance in modeling longitudinal EAT changes, supporting their suitability for early-phase, exploratory imaging research.

While these findings demonstrate the feasibility of AI-based modeling of longitudinal CT-derived epicardial adipose tissue changes, their clinical implications remain exploratory. The results should be interpreted as hypothesis-generating, supporting further validation in larger, multicenter cohorts before integration into routine clinical workflows.

Future studies involving larger, multicenter cohorts and clinical outcome data are warranted to validate these results and to further explore the clinical and diagnostic implications of AI-assisted longitudinal EAT assessment.

## Figures and Tables

**Table 1:** Pre–Post Comparison of Adipose Tissue Compartments.

Variable	Mean Δ	Paired *t*-Test (*p*)	Wilcoxon Test (*p*)
Epicardial adipose tissue (mL)	+23.8	0.0010	0.0011
Visceral fat volume (mL)	+318.5	0.0374	0.0512

**Table 2:** Linear Regression Analysis for Absolute ΔEAT (*n* = 42).

Variable	Direction of Association	Relative Contribution
Follow-up duration	Positive	Highest
Dyslipidemia	Positive	Moderate
Baseline EAT volume	Negative	Low
Sex	Negative	Low
Diabetes mellitus	Negative	Low
Age	Negative	Low
Body mass index	Neutral	Minimal

**Table 3:** Linear Regression Analysis for Normalized ΔEAT per Month (*n* = 13).

Variable	Direction of Association	Relative Contribution
Baseline EAT volume	Positive	Highest
Arterial hypertension	Positive	High
Body mass index	Negative	Moderate
Diabetes mellitus	Negative	Moderate
Dyslipidemia	Negative	Moderate
Follow-up duration	Neutral	Minimal

**Table 4:** Performance metrics of AI models for classification of EAT increase (ΔEAT > 0).

Model	AUC	Accuracy	F1-Score
SVM	0.817	0.762	0.862
ANN (LBFGS)	0.900	0.917	0.944
XGBOOST	0.500	0.488	0.455

**Table 5:** Summary of longitudinal changes in epicardial adipose tissue and model performance.

Domain	Metric	Result
EAT longitudinal change	Mean ΔEAT (mL)	+23.8
	Median ΔEAT (mL)	+26.0
	95% CI of mean ΔEAT (mL)	10.2–37.4
	Paired *t*-test (*p*-value)	0.0010
	Wilcoxon test (*p*-value)	0.0011
Visceral fat change	Mean Δ visceral fat (mL)	+318.5
	Paired *t*-test (*p*-value)	0.0374
	Wilcoxon test (*p*-value)	0.0512
AI classification	Best-performing model	ANN (LBFGS)
	AUC (best model)	0.90
	Accuracy (best model)	0.92
	F1-score (best model)	0.94

## Data Availability

The original contributions presented in this study are included in the article. Further inquiries can be directed to the corresponding author.
